# Dr. Edson H. Young

**Published:** 1905-03

**Authors:** 


Dr. Edson H. Young, of Buffalo, a graduate of the medical department of the University of Vermont, 1884, died at his home, February 5, 1905, aged 44 years. Dr. Young was a conscientious, active physician who led a busy life and had a large professional following. He died after a week's illness in the midst of a useful professional life.

